# Primary Thyroid Extranasal NK/T-Cell Lymphoma Associated With Good Outcome: A Case Report and Literature Review: A Care-Compliant Article

**DOI:** 10.1097/MD.0000000000003460

**Published:** 2016-05-20

**Authors:** Jun-he Li, Hong-hong He, Yuan Cheng, Wen-jing He

**Affiliations:** From the Department of Oncology (J-HL, H-HH, YC), First Affiliated Hospital of Nanchang University, Nanchang; Department of Endocrinology (W-JH), First Affiliated Hospital of Sun Yat-sen University, Guangzhou, China.

## Abstract

Most thyroid lymphomas are B-lineage, and T-cell lymphomas are rare. None of primary thyroid extranasal NK/T-cell lymphoma (NKTCL) has been reported in the literature. Here, we report a case of extranasal NKTCL exclusively arising in the thyroid in an 18-year-old Chinese.

The patient presented with rapid anterior swelling at the neck and aggravated dyspnea for 2 months. Neck computer tomography scan revealed diffuse thyroid enlargement in the left lobe compressing the trachea. The thyroid function test was indicative of hypothyroidism. Gastroscopy demonstrated chronic nonspecific gastritis. Subtotal thyroidectomy was performed. Histological examination showed a diffuse infiltration of neoplastic lymphoid cells with an angiodestructive behavior. Immunophenotype is positive for CD2, CD56, CD43, and TIA-1, and typically negative for surface CD3. Epstein-Barr virus-encoded small RNAs were detected in tumor cells. A diagnose of primary thyroid extranasal NKTCL-N lymphoma was confirmed by the findings.

The patient was treated with CHOP-L combination chemotherapy followed by local radiotherapy, and tolerated the modality well. The patient has been in remission for 28 months so far.

To our knowledge, this is the first case report of primary extranasal NKTCL exclusively arising in the thyroid. The case has a relatively good treatment outcome with timely diagnosis and multimodality approach.

## INTRODUCTION

Primary thyroid lymphomas (PTL) are defined as a lymphomatous process involving the thyroid gland without contiguous spread or distant metastases from other areas of involvement at diagnosis.^1^ PTL is rare, accounting for *<*1% cases of extranodal lymphomas. Pathologically, most thyroid lymphomas are non-Hodgkin lymphomas of B-cell origin with Hodgkin and T-cell thyroid lymphomas occurring rarely.^2–4^ Our review of the medical literature revealed none of primary thyroid extranasal NK/T-cell lymphoma (NKTCL) published so far. Here, we report the first case of primary thyroid NKTCL associated with a good outcome and discuss the clinical features, treatment, and prognosis.

### Case Report

#### Clinical Findings

An 18-year-old man was admitted for rapid anterior swelling at the neck with aggravated dyspnea for 2 months, hoarseness, irritability restlessness, recurrent fever, and 6-kg weight loss were also present. He had neither previous nor family history of thyroid disease. Physical examination revealed a large, hard thyroid mass in the left lobe with deviation of the laryngeal cartilage, and the patient indicated feeling oppression of the neck. Laboratory tests were as follows: white blood cell count 8.07 × 10^9^ cells/L, red blood cell count 5.47 × 10^12^ cells/L, hemoglobin level 100 g/L, and a platelet level 216 × 10^9^ cells/L. Thyroid function test indicated hypothyroidism (thyroid-stimulating hormone (TSH) 77.64 m unit/mL, free T3 2.53 ng/dL, free T4 13.69 ng/dL). Thyroglobulin (Tg) and antimicrosome antibody were within normal limits. The serum lactate dehydrogenase (LDH) level was 426 U/L (normal range 114–240 U/L). He also had elevated AST/ALT about 3 times the normal upper limit, EBV IgG is positive.

**FIGURE 1:**
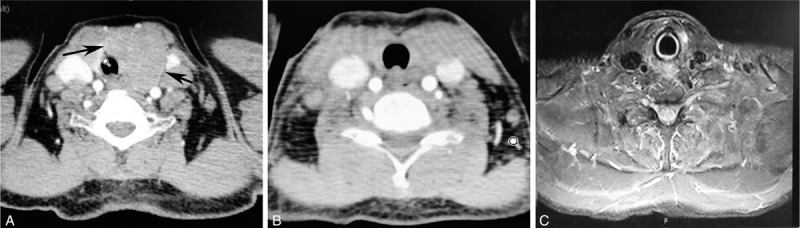
Computed tomography (CT) scans of the neck. CT picture before subtotal thyroidectomy showed diffuse thyroid enlargement in the left lobe compressing the trachea (A, black arrow). CT picture after subtotal thyroidectomy (B) and the most recent MRI picture (C) revealed no local recurrence.

Neck computer tomography scan revealed diffuse thyroid enlargement in the left lobe compressing the trachea (Figure 1A). Gastroscopy with random biopsies demonstrated chronic nonspecific gastritis; nasal endoscopy did not find lesions in the nasal cavities. CT scans of the head, chest, and abdomen did not detect enlarged lymph nodes. The liver and spleen were of normal size and shape.

Written informed consent was obtained from the patient's direct relative for publication of this case report and related images. Ethics approval has been obtained from the Human Ethics Committee of The First Affiliated Hospital, Sun Yat-sen University and the First Affiliated Hospital of Nanchang University.

#### Pathologic Findings and Treatment

**FIGURE 2:**
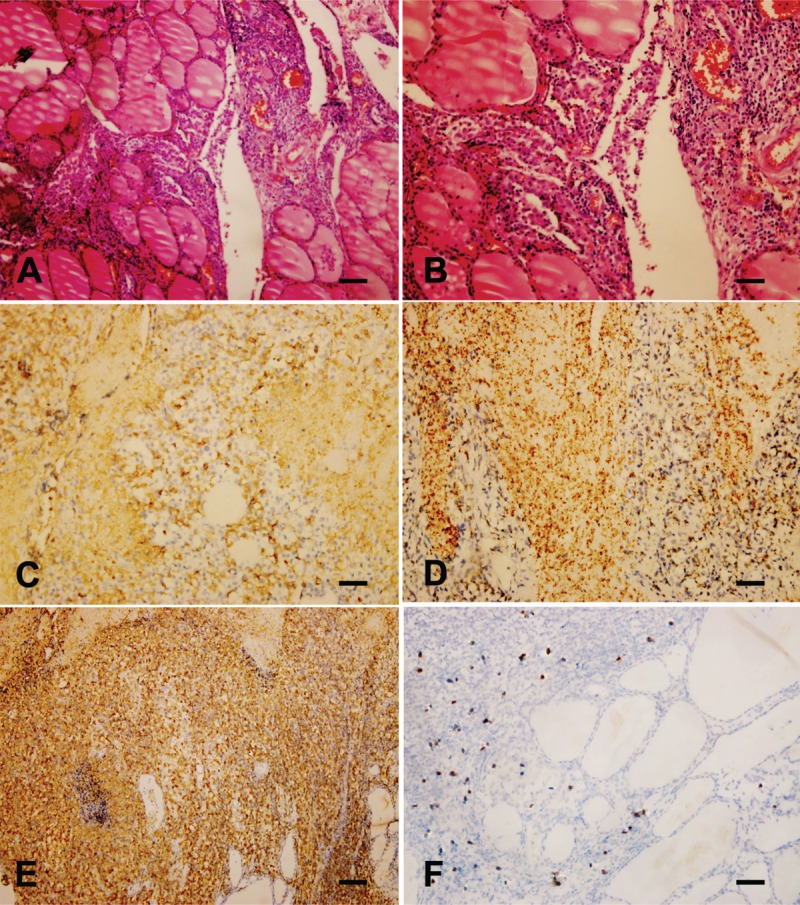
Pathological features of postoperative specimen. Histological examinations stained with hematoxylin and eosin revealed diffuse infiltration of small to medium-sized lymphoid cells with angiodestructive growth pattern, regions of necrosis, and apoptotic bodies (A and B). The tumor cells were positive for CD56 (C), LCA (D), TIA-1 (E), and EBER (F), bars = 50 um.

Symptoms were severe; therefore, the subtotal thyroidectomy was performed to obtain a definite diagnosis and relief of the local symptoms caused by an enlarged goiter. CT pictures after subtotal thyroidectomy are shown in Figure 1B. Histological examination showed diffuse infiltration of small to medium-sized lymphoid cells with angiodestructive growth pattern, and some admixed mitotic figures, regions of necrosis, and apoptotic bodies were present (Figure 2A and B). The tumor cells were positive for CD2, CD43, CD56 (Figure 2C), LCA (Figure 2D), and TIA-1 (Figure 2E), and negative for surface CD3. In situ hybridization (ISH) for EBER showed positive signals in tumor cells (Figure 2F). T-cell receptor gene rearrangements were not detected with cytogenetic analysis. A diagnosis of primary thyroid extranasal NKTCL was confirmed. Staging procedures did not reveal any other involvement (stage IE).

**FIGURE 3:**
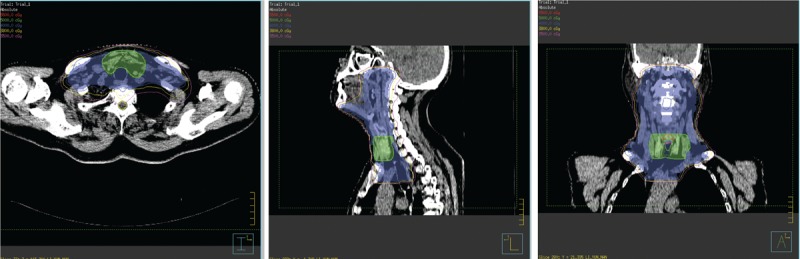
Pictures of the radiotherapy fields and target volumes.

Ten days after surgery, the patient was offered combined chemotherapy followed by radiotherapy. CHOP-L regimen (L-asparaginase, cyclophosphamide, doxorubicin, vincristine, and prednisone) was given every 3 weeks, 6 cycles in total. The patient received intensity-modulated radiotherapy (IMRT) by using 6MV photons, CTV1 consisted of cervical and upper mediastinal lymph node regions, and CTV2 was defined as preoperative tumor bed (PTV), PTV included CTV and a 5-mm margin. The total dose of PTV1 and PTV2 was 50 Gy in 24 fractions at 2.08 Gy and 40 Gy in 24 fractions at 1.67 Gy respectively. The radiotherapy fields and target volumes are shown in Figure 3. The patient tolerated well, and the adverse reactions were grade 1 to 2 myelosuppression and digestive tract toxicities. The patient was monitored regularly with computed tomography or magnetic resonance imaging (MRI).The most recent neck MRI pictures (Figure 1C) and chest and abdominal CT scan revealed no evidence of recurrence. The patient has been in remission for 28 months until now.

## DISCUSSION

Reviewing the literature, primary thyroid lymphoma occurs most frequently in women older than 50 years. Patients often had a past history of Hashimoto thyroiditis, and some of them, thyroid function tests were indicative of hypothyroidism.^3,5,6^ Most patient presented a rapidly enlarging, painless thyroid mass, often accompanied by pressure symptoms from the aerodigestive tract, including hoarseness, dysphasia, and dyspnea.^5–7^ “B” symptoms were less common. Most reported cases were classified as B-cell lymphomas, which included diffuse large B-cell lymphoma (DLBCL) and mucosa-associated lymphoid tissue (MALT) lymphoma. Open biopsy or surgery was the most common method for diagnosis. Treatment included chemotherapy, with or without surgery, and radiotherapy, but no consensus has been reached.^2–8^ Primary thyroid lymphoma is a disease with a relatively good prognosis. In the literature, the reported 5-year overall survival rates of primary thyroid lymphoma ranged from 66% to 100%.^4–11^ This case is a male adolescent without a history of Hashimoto thyroiditis, and he had “B” symptoms. These characteristics were different from those of the usual thyroid lymphoma (Table 1).

**TABLE 1:**
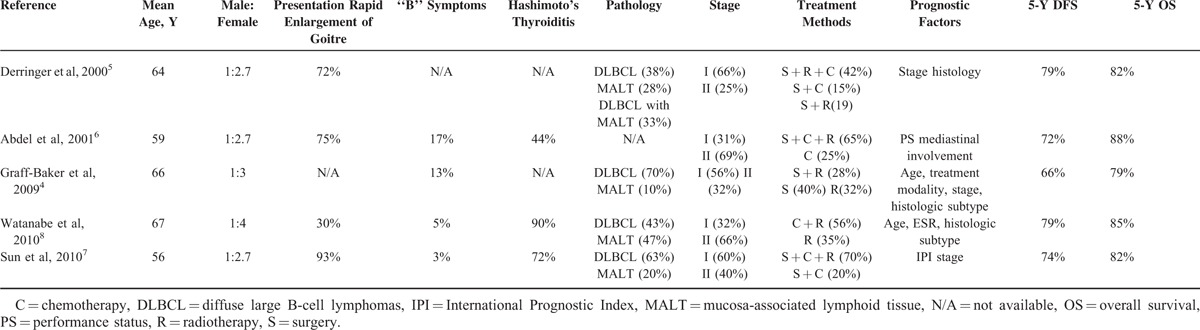
Lists of Some Reported Thyroid Lymphoma

The World Health Organization (WHO) classification groups both nasal and extranasal NKTCL lymphoma in the same category as “nasal type.” According to WHO classification of hematolymphoid tumors, this case belongs to extranasal NKTCLs.^12^ Any anatomic site may be involved at the disease presentation or during disease progression. Involvement of the skin, the testis, the gastrointestinal tract, the eyes, the lungs, the adrenal glands, the soft tissues, the breast, the brain, and the tongue has been reported.^13–15^ Morphologically, the disease is characterized by a diffuse infiltration of neoplastic lymphoid cells with an angiodestructive behavior. The neoplastic cells express LCA and CD56 and are negative for surface CD3, and EBV is almost always expressed within the neoplastic cells.^16^ The characteristics of the NKTCL in our patient were typical including EBV positivity and tissue biopsy characteristics. Patients with extranasal NKTCL often have more adverse clinical features such as poor performance status and an advanced stage, and are more likely to have cytopenias, when compared with patients with nasal lymphoma.^16,17^ Although the case in this report had “B” symptoms and elevated LDH, he had an early stage, and the performance status was not too bad, which is inconsistent with the previously published data.

Due to the extremely low incidence of the disease, the strategy for management of extranasal NKTCL remains controversial and refers to those of nasal-type NKTCL. Systemic chemotherapy and involved field radiotherapy is the current suggestion for management of nasal-type NKTCL.^18^ Radiotherapy is important for localized nasal disease.^19,20^ Conventional chemotherapy regimens for extranodal NKTCL do not differ for nasal and extranasal disease, and CHOP is a very common regimen.^21^ Few survival statistics exist for extranasal NKTCL. In a recent international multicenter study reported by the International Peripheral T-cell Lymphoma Project, the median overall survival was worse in extranasal compared with the nasal cases in earty-stage (0.36 vs 2.96 years) and late-stage disease (0.28 vs 0.8 years).^22^ The treatment outcome is far from satisfactory. For patients with extranasal disease, new treatments are clearly needed. The introduction of L-asparaginase-containing regimens led to further improvements, as most studies using L-asparaginase-based regimens in a relapsed or refractory setting reported response rates of around 50 %.^23–25^ A prospective phase II study also demonstrated that CHOP-L in combination with radiotherapy was excellent for newly diagnosed ENKTL.^26^ Therefore, the use of L-asparaginase-based regimens may be superior to the use of CHOP regimen alone.

Age, B symptoms, local tumor invasion, clinical stage, CR rate, NK/T-cell PI, LDH and EBV infection were reported to have prognostic significance in ENKTL lymphoma.^20,21,27,28^ Although our patient had unfavorable factors (elevated lactate deshydrogenase, EBV infection, and “B” symptoms), he has been in remission for 28 months so far. The good treatment outcome may be contributed to the early stage, the thyroid involvement and timely multimodality approach. The experience is from only 1 case, but we hope that awareness of this entity will help oncologists achieve timely diagnosis and intervention.

## CONCLUSION

In conclusion, we report the first case of primary extranasal NKTCL. The case had clinical features different from those of the usual thyroid lymphoma and extranasal NKTCLs arising from other sites. The patient had relatively good treatment outcome with timely diagnosis and multimodality approach.
